# Vitamin C as a Supplementary Therapy in Relieving Symptoms of the Common Cold: A Meta-Analysis of 10 Randomized Controlled Trials

**DOI:** 10.1155/2020/8573742

**Published:** 2020-10-09

**Authors:** Li Ran, Wenli Zhao, Hongwu Wang, Ye Zhao, Huaien Bu

**Affiliations:** ^1^Graduate School, Tianjin University of Traditional Chinese Medicine, Tianjin 300193, China; ^2^Department of Occupational and Environmental Health, School of Health Sciences, Wuhan University, Wuhan 430071, China; ^3^Liver Center, Saga University Hospital, Saga University, 849-8501, Japan; ^4^School of Health science and Engineering, Tianjin University of Traditional Chinese Medicine, Tianjin 300193, China; ^5^Qingdao Academy of Traditional Chinese Medicine, Shandong University of Traditional Chinese Medicine, Qingdao 266112, China

## Abstract

**Aim:**

To investigate whether vitamin C performs well as a supplemental treatment for common cold.

**Method:**

After systematically searching through the National Library of Medicine (PubMed), Cochrane Library, Elsevier, China National Knowledge Infrastructure (CNKI), VIP databases, and Wanfang databases, 10 randomized controlled trials were selected for our meta-analysis with RevMan 5.3 software. Published in China, all 10 studies evaluated the effect of combined vitamin C and antiviral therapy for the treatment of common cold.

**Results:**

The total efficacy (RR = 1.27, 95% CI (1.08, 1.48), *P* = 0.003), the time for symptom amelioration (MD = −15.84, 95% CI (-17.02, -14.66), *P* < 0.00001), and the time for healing (*I*, 95% CI (-14.98, -4.22), *P* = 0.0005) were better with vitamin C supplementation than with antiviral therapy alone.

**Conclusions:**

Vitamin C could be used as a supplementary therapy along with antiviral regimens to relieve patients from the symptoms of common cold.

## 1. Introduction

Common cold (also known as cold), which is a common and frequent disease, is an upper respiratory tract infection (URTI) mainly caused by viruses that includes uncomfortable symptoms like stuffy or runny nose, coughing or sneezing, sore throat, chills, fever, and headache [1, 2].

Common cold is self-limited and lasts between seven and ten days [3]. Generally, it is unnecessary to administer any antiviral medicine to treat cold, for the symptoms are usually mild and there is still no effective antiviral treatment [2]; however, when it progresses quickly and presents with severe symptoms, over-the-counter (OTC) medications (nasal decongestants, antihistamines, cough suppressants, nonsteroidal antipyretic analgesics, and expectorants) should be promptly administered to relieve patients from symptoms. Although common cold itself is not a threat under normal circumstances, it still imposes a substantial economic and societal burden in two ways.

The incidence of common cold is high. As indicated in some reports, children suffer an average of 6 to 10 colds per year (up to 12 colds for school children), in contrast to adults, who experience only 2 to 5 colds annually, each lasting an average of 9 days [4, 5]. Due to the high incidence, absence from work and reduction of work capacity caused by common cold inflict great economic damage. Sweden bore an estimated annual total productivity loss of 2.7 billion Euros (US $3.17 billion), and the mean loss of productive capacity was at 5.1 days per employee per year [6]; in addition, in the United States, 22 to 189 million school days and 20 to 126 million workdays are lost annually [7, 8], and the economic burden is approximately $40 billion (95% confidence interval, $31.2 to $48.0 billion) [9]. After common cold infection, physician visits and prescription medications resulted not only in unnecessary medical waste but also in increased economic pressures. Before 2000, approximately 27 million physician visits were made annually, and the cost to purchase OTCs for common cold and other viral respiratory infections was almost $2 billion; in 2013, the cost of OTCs for common cold alone was up to $2.9 billion dollars [10]. According to the data from the World Health Organization (WHO) in 2013, common cold led to 75 to 100 million physician visits at a conservative cost of $7.7 billion per year in the United States, of which, $400 million were spent for prescription medications. At the same time, in the United Kingdom, the medical cost of OTCs for coughs and cold reached 500 million pounds ($644.35 million) per year [11]. While there is no economic loss estimation due to common cold in China, consumption of cold medications in the retail market reached 9.2 billion Chinese Renminbi (RMB, US $379.08 million) in 2008 alone [12].

Symptoms such as fever, cough, and headache usually subside within a few days without any serious complications; however, severe colds, particularly comorbid with meningitis or lower respiratory tract infection (LRTI) such as pneumonia could be fatal if untreated, especially for individuals with compromised immune system [13]. Common cold (or respiratory infection) is the root of all diseases, which suggests that untreated cold may lead to LRTIs and diseases in other organs. According to the statistics in China, there is a close relationship between common cold and the onset, aggravation, and relapse of chronic bronchitis—an estimated 56.4% of new chronic bronchitis cases have a history of cold, and 65.4% of acute chronic bronchitis attacks are associated with cold. In addition, almost one-third of pneumonias develop from cold [14]. Additionally, 5% of the seasonal increase in mortality associated with cardiovascular disease is related to respiratory infection [15, 16].

Over 200 serologically different viral strains are responsible for human URTIs, with rhinoviruses being the most common [17]. Although 70% to 80% of common cold cases are caused by viruses, there is still a small percentage caused by bacteria. As a result, we consider a misuse of antibiotics and antiviral therapy in treating the common cold or URTIs clinically [18]. Through this meta-analysis, we aim to evaluate the efficacy of vitamin C treatment and advocate for a rational use of medicine.

## 2. Methods

### 2.1. Search Strategy

Two independent reviewers systematically searched in PubMed, Cochrane Library, Elsevier, China National Knowledge Infrastructure (CNKI), VIP databases, and Wanfang databases, from their earliest records through December 2019. The following key words were used: common cold, URTI, Vitamin C, and ascorbic acid. Subsequently, studies were screened and selected according to the guidelines for Meta-Analyses and Systematic Reviews of Observational Studies (MOOSE) [19] and Preferred Reporting Items for Systematic Reviews and Meta-Analyses (PRISMA) [20].

### 2.2. Inclusion Criteria

#### 2.2.1. Study Pattern

Using vitamin C as a therapeutic technique with antiviral therapies, all randomized controlled trials (RCTs) related to common cold were included in our study. Studies were published in English or Chinese.

#### 2.2.2. Study Participants

We selected study participants who were diagnosed with common cold based on laboratory examination, clinical signs, and reported symptoms. More specifically, common cold is presenting with catarrhal symptoms, such as stuffy nose, runny nose, sneezing, and coughing. Then, identification of viruses and exclusion of other pathogenesis was conducted by viral culture, antigen detection, and polymerase chain reaction (PCR).

#### 2.2.3. Intervention

Through literature search, we detected a trend of prescribing antiviral drugs in China, unlike Europe and the United States, which may be caused by differences in patient health and national medical practices. Generally speaking, almost all RCTs were conducted comparing vitamin C and placebo in Europe and the United States, while the others were comparing vitamin C in combination with antiviral therapies versus antiviral therapies alone in China. Based on the distinct intervention that we regarded as an explanation possibly for the strong clinical heterogeneity, the treatment group must have received intervention with vitamin C as well as antiviral therapy, while the control group received antiviral therapy only.

#### 2.2.4. Outcome

*(1) Efficacy Criteria*. The efficacy criteria were (1) total effective rate, (2) average time for symptom amelioration, and (3) average time for healing.

*(2) Adverse Events*. Any abnormal signs and symptoms during treatment were regarded as adverse events.

### 2.3. Exclusion Criteria

Exclusion criteria were (1) duplicated articles; (2) nonclinical trials, such as animal testing; (3) non-RCTs, such as case reports and experiences, theory research, and reviews; (4) the assessment of vitamin C in the prevention of common cold; and (5) treatment group did not undergo combination therapy with vitamin C and antiviral medication.

### 2.4. Quality Assessment

Quality assessment was performed on the following items according to Cochrane Collaboration's tool for bias risk: (1) random sequence generation, (2) allocation concealment mechanism, (3) blinding of participants and personnel, (4) blinding of outcome assessment,(5) incomplete outcome data, (6) selective reporting, and (7) other biases. The outcomes were independently evaluated by two researchers as high risk, unclear, and low risk.

### 2.5. Statistical Analysis

For the “incidence” parameters like efficacy and adverse events, the total number of participants and different events in the treatment and control groups were extracted. However, for the “severity” parameters, such as average time for symptom amelioration and average time for healing, the mean values with standard deviation (SD) were calculated. All parameters were entered in Review Manager 5.3 software for analysis with the corresponding 95% confidence intervals (CI).

Heterogeneity was detected by the Chi-squared test and inconsistency- (*I*-) squared statistics. In our meta-analysis, the random-effects model was used when *P* ≤ 0.05 or *I*^2^ > 50%; otherwise, the fixed-effects model was preferred.

## 3. Results

### 3.1. Study Selection

**Figure 1:**
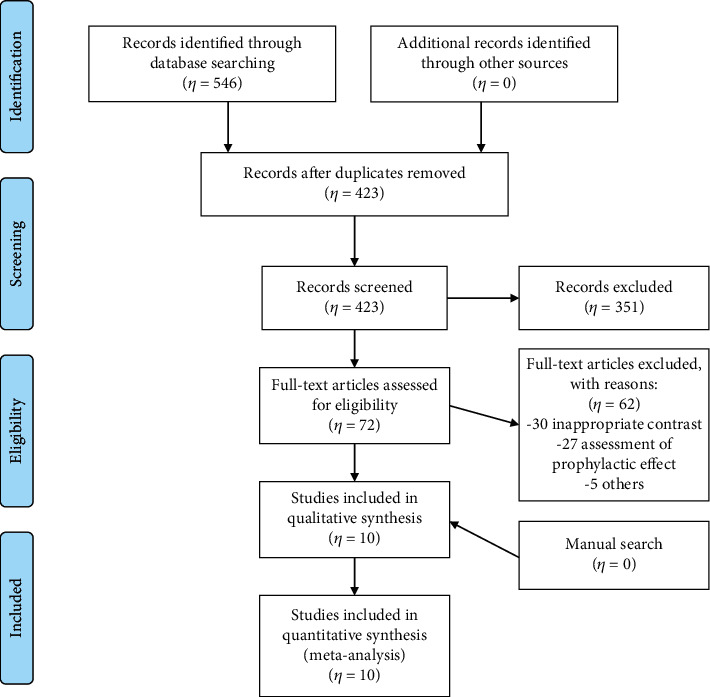
Study selection procedure.

Of the 546 RCTs, 123 duplicate trials identified in the PubMed, Cochrane Library, Elsevier, CNKI, VIP, and Wanfang data were excluded. Additional 351 RCTs were excluded based on titles and abstracts, and 62 were excluded after full-text review. The study selection procedure is outlined in Figure 1.

### 3.2. Study Characteristics

As shown in Table 1, the 10 selected RCTs recruited patients that were treated for common cold with a combination of vitamin C and antiviral therapy or antiviral therapy alone. These studies included 1048 patients and were conducted between 2007 and 2019.

### 3.3. Quality of the Included Studies

Table 2 shows the quality of included studies, with the risk of bias based on the Cochrane Handbook. All studies were 100% completed and had no bias in selective reporting, but none had any description about allocation concealment, blinding of participants and personnel, blinding of outcome assessment, or other bias. Only three articles [23, 25, 29] demonstrated that a random sequence was generated with random number tables, and there was no detailed information in the remaining seven.

### 3.4. Meta-Analysis of Outcome Criteria

#### 3.4.1. Total Effective Rate

**Figure 2:**
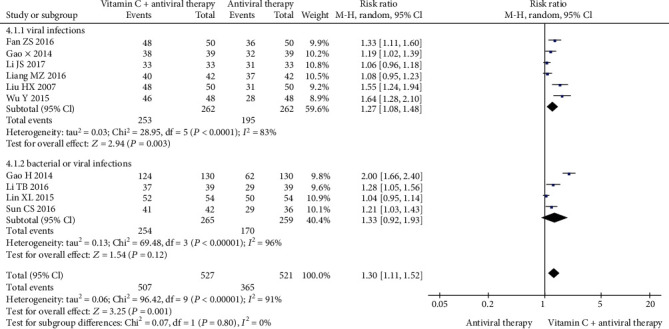
Forest plot of the meta-analysis of total efficacy.

The total effective rate was reported in 10 RCTs [21–30] in China, involving 1048 participants. We performed subgroup analyses to make sense whether the effective rate is different between viral infections and unexplained infections (bacterial or viral infection). According to the outcomes shown in Figure 2, vitamin C combined with antiviral therapy was significantly more effective than antiviral therapy alone in viral infections (RR = 1.27, 95% CI (1.08, 1.48), *P* = 0.003); vitamin C combined with antiviral therapy showed no difference to antiviral therapy alone in bacterial or viral infections (RR = 1.33, 95% CI (0.92, 1.93), *P* = 0.12).

#### 3.4.2. Average Time for Improving

**Figure 3:**
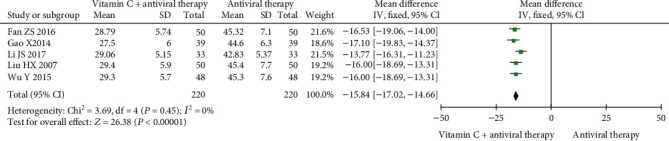
Forest plot of the meta-analysis of average time for symptom amelioration.

The average time for symptom amelioration was reported in five trials [21, 23–25, 27] involving 440 patients. No heterogeneity was observed (Cochrane *Q* test = 3.69, df = 4, *P* = 0.45, *I*^2^ = 0%), and the vitamin C group supplementation performed better (MD = −15.84, 95% CI (-17.02, -14.66), *P* < 0.00001) than the antiviral therapy alone group (Figure 3).

#### 3.4.3. Average Time for Healing

**Figure 4:**
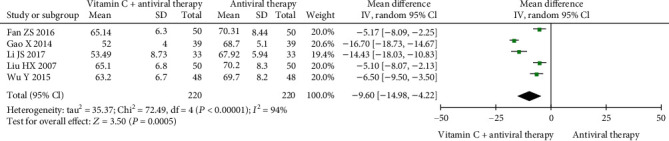
Forest plot of the meta-analysis of average time for healing.

The average time for healing was reported in five trials [21, 23–25, 27] involving 440 patients. Due to high heterogeneity (Cochrane *Q* test = 72.49, df = 4, *P* < 0.00001, *I*^2^ = 94%), meta-analysis was performed using the random-effects model, showing a significant difference (MD = −9.60, 95% CI (-14.98, -4.22), *P* = 0.0005) between the vitamin C supplementation group and the antiviral therapy alone group. (Figure 4).

#### 3.4.4. Adverse Events

Three of the 10 studies reported adverse events. There was no statistical difference between the treatment group and the control group (*P* = 0.18).

Li and Wang [28] showed that the incidence of mild diarrhea was 2.56% in the treatment group, while the incidence of anorexia and mild diarrhea was 7.69% in the control group (*P* > 0.05).

There were two cases of rash and three cases of pruritus in the control group, and two cases of rash as well as one case of soreness in the treatment group. The incidence rate of adverse events was 11.90% and 7.14%, respectively, with no statistical significance (*P* > 0.05) [29].

As reported in Fan's study [21], one case of rash occurred with antiviral therapy alone.

#### 3.4.5. Funnel Plot

**Figure 5:**
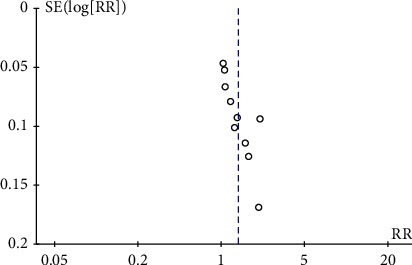
Funnel plot of publication bias for the comparison of vitamin C plus antiviral therapy with antiviral therapy alone.

Visual inspection of the funnel plot was conducted to detect publication bias of all studies. Each study was represented by a circle, while vertical lines represented the pooled effect. There was a publication bias, as summarized in Figure 5.

## 4. Discussion

### 4.1. Efficacy Assessment of Vitamin C

We show strong evidence that vitamin C is important for the prevention and treatment of common cold. Vitamin C helps to relieve patients from coughing and catarrh symptoms, through its antihistaminic action, decrease of 5-hydroxytryptamine, or synthesis of prostaglandins [24, 31–33]; in addition, vitamin C stimulates and enhances immunological functions and assists to virus killing in the pathways by scavenging free radicals and supplying energy for cells [34–38].

Based on immunity, vitamin C contributes to the prevention and treatment of common cold in four ways. First, vitamin C is a constituent of white blood cells, which fight diseases through engulfment and phagocytosis of pathogens. During common cold, supplementation with vitamin C markedly increases the capability to resist pathogenic bacteria and shortens the disease course [39, 40]. Second, adequate vitamin C can assist with the production of nutrients such as proteins, lipids, selenium, vitamin A, and vitamin E, through reduction reaction. Nutrients then support production and activity of antibodies and ensure proper metabolism of cells [41]. In addition, vitamin C participates in specific immune responses with two different approaches, namely, T-cell-mediated immunity and B-cell-mediated humoral immunity [42–46]. Furthermore, C1 synthesis might be associated with serum vitamin C levels, whereas synthesized C1q molecules induce the classical pathway of complement activation after binding to IgGC_H_2 and IgMC_H_3 domains, thereby enabling nonspecific immune defense [47]. Last but not the least, vitamin C enhances immunity through phagocyte activation. The effects of vitamin C on phagocytes are exerted through three different mechanisms: direct impact on phagocytosis, influence on cell migration and chemotaxis, and protection of phagocytes [48, 49].

In our meta-analysis, the total efficacy (RR = 1.27, 95% CI (1.08, 1.48), *P* = 0.003), the time for symptom amelioration (MD = −15.84, 95% CI (-17.02, -14.66), *P* < 0.00001), and the time for healing (MD = −9.60, 95% CI (-14.98, -4.22), *P* = 0.0005) were all better after vitamin C supplementation than after antiviral therapy alone. Due to the high heterogeneity in the comparisons of efficacy and healing time, we cannot safely conclude that vitamin C is a complementary treatment to antiviral therapy, although it is clear that it helps to improve cold symptoms. More high-quality RCTs are required in the future, to prove the efficacy of vitamin C supplementation in treating common cold.

### 4.2. Rational Use of Medications

During the process of literature selection and data analysis, we revealed an interesting phenomenon: patients with mild cold in Europe and the United States are requested to rest in bed, drink fluids, and supplement with vitamin C, while patients in China tend to be prescribed antiviral drugs and even antibiotics rather than vitamin C.

An investigation conducted by the Chinese Asthma Alliance and Evidence-Based Center of China in 2010 indicated that the knowledge on the treatment of common cold varied among clinics, due to the varying levels of healthcare and education in different areas of China. When common cold occurs, antibiotics may be the first choice to resolve inflammation and relieve patients from symptoms. The irrational use of antibiotics occurs especially in primary hospitals and in children [50–52]. Since evidence has demonstrated that antibiotics are ineffective for acute URTI (AURTI) and common cold [53–55], antibiotics are not recommended [56–58].

Additionally, misinterpretation of the function and usage of ribavirin may occur in some clinics. Ribavirin is generally regarded as an antiviral therapeutic for the treatment of AURTI in China [59]; however, it is strictly limited to the treatment of chronic hepatitis C and children's severe LRTIs caused by respiratory syncytial virus [59–62]. The newly published “Essential Medicines List” (version 2017) [63] summarizes clear guidelines for ribavirin, which is typically used for the treatment of viral hemorrhagic fevers and hepatitis C.

Finally, irrational use of the combinations of OTC cold medications often occurs, due to the lack of medication guidance or elementary knowledge of hygiene, thus potentially leading to increased costs, drug interactions, more side effects, and administration of unnecessary drugs [15, 64, 65].

The following advice is recommended for the treatment of cold: (1) OTC medications are recommended for relieving patients from symptoms instead of antiviral therapy [66] (for example, a first-generation antihistamine and decongestant preparation is recommended for patients with acute cough associated with common cold [56, 67]); (2) oral medicine, rather than intravenous infusion, is the first treatment choice [66]; (3) antibiotics should be used only when there is evidence of secondary bacterial infection; (4) cough and cold medicine for young children should include acetaminophen or ibuprofen instead of aspirin, due to risk of Reye's syndrome; packaging of cough and cold medicines for young children should display a warning similar to that recommended by the US Food and Drug Administration [68]; and (5) traditional Chinese medicine is recommended [69].

### 4.3. Limitation

There were some limitations to our study. First, the 10 RCTs comparing antiviral therapy alone with vitamin C supplementation should have been more normative and clear to minimize bias. There was no description of the generation of random sequence in most trials, and it was not discussed whether the studies were double-blinded or whether allocation was concealed. We sincerely advocate for standardization of study theses and increased efforts to improve the quality of methodologies. Furthermore, publication bias may result in misleading outcomes.

## 5. Conclusion

Supplementation with vitamin C during the treatment of common cold could benefit symptom improvement.

## Figures and Tables

**Table 1:** Characteristics of included studies for the comparison of vitamin C plus antiviral therapy with antiviral therapy alone.

Author	Year	Sample size (T/C)	Gender (male/female)		Age (T/C, year)	Intervention		Outcome
			Treatment	Contrast		Treatment	Contrast	
Fan [21]	2016	50/50	Not reported	Not reported	Not reported	Antiviral therapy + vitamin C	Antiviral therapy	Total efficacy + symptom improvement + side effects
Gao and Wang [22]	2014	130/130	69/61	72/58	Not reported	Antiviral therapy+ vitamin C	Antiviral therapy	Total efficacy
Li [23]	2017	33/33	15/18	16/17	21.84 ± 1.02/21.68 ± 1.10	Antiviral therapy + vitamin C	Antiviral therapy	Total efficacy + symptom improvement
Wu [24]	2015	48/48	Not reported	Not reported	3.4 ± 1.1/3.2 ± 0.9	Antiviral therapy + vitamin C	Antiviral therapy	Total efficacy + symptom improvement
Gao [25]	2014	39/39	Not reported	Not reported	3.5 ± 1.0	Antiviral therapy + vitamin C	Antiviral therapy	Total efficacy + symptom improvement
Sun [26]	2016	42/36	27/15	Not reported	41.7 ± 3.5/40.0 ± 3.8	Antiviral therapy + vitamin C	Antiviral therapy	Total efficacy
Liu [27]	2007	50/50	31/19	28/22	Not reported	Antiviral therapy + vitamin C	Antiviral therapy	Total efficacy + symptom improvement
Li and Wang [28]	2016	39/39	22/17	21/18	39 ± 2.63/38 ± 2.14	Antiviral therapy + vitamin C	Antiviral therapy	Total efficacy + side effects
Liang [29]	2016	42/42	22/20	24/18	36.8 ± 8.1/37.1 ± 7.5	Antiviral therapy + vitamin C	Antiviral therapy	Total efficacy + side effects
Lin [30]	2015	54/54	35/19	34/20	38.9 ± 15.8/38.3 ± 16.9	Antiviral therapy + vitamin C	Antiviral therapy	Total efficacy + side effects

**Table 2:** Cochrane Collaboration's tool for assessing the risk of bias for the comparison of vitamin C plus antiviral therapy with antiviral therapy alone.

Studies	Random sequence generation	Allocation concealment	Blinding of participants and personnel	Blinding of outcome assessment	Incomplete outcome data	Selective reporting	Other bias
Fan 2016 [21]	Unclear	Unclear	Unclear	Unclear	Low risk	Low risk	Unclear
Gao and Wang 2014 [22]	Unclear	Unclear	Unclear	Unclear	Low risk	Low risk	Unclear
Li 2017 [23]	Low risk	Unclear	Unclear	Unclear	Low risk	Low risk	Unclear
Wu 2015 [24]	Unclear	Unclear	Unclear	Unclear	Low risk	Low risk	Unclear
Gao 2014 [25]	Low risk	Unclear	Unclear	Unclear	Low risk	Low risk	Unclear
Sun 2016 [26]	Unclear	Unclear	Unclear	Unclear	Low risk	Low risk	Unclear
Liu 2007 [27]	Unclear	Unclear	Unclear	Unclear	Low risk	Low risk	Unclear
Li and Wang 2016 [28]	Unclear	Unclear	Unclear	Unclear	Low risk	Low risk	Unclear
Liang 2016 [29]	Low risk	Unclear	Unclear	Unclear	Low risk	Low risk	Unclear
Lin 2015 [30]	Unclear	Unclear	Unclear	Unclear	Low risk	Low risk	Unclear

## Data Availability

Data are all contained within the paper.

## Abbreviations

PubMed: National Library of Medicine
CNKI: China National Knowledge Infrastructure
URTI: Upper respiratory tract infection
LRTI: Lower respiratory tract infection
AURTI: Acute upper respiratory tract infection
MOOSE: Systematic Reviews of Observational Studies
PRISMA: Preferred Reporting Items for Systematic Reviews and Meta-Analyses
RCTs: Randomized controlled trials
PCR: Polymerase chain reaction
SD: Standard deviation
RR: Risk ratio
CI: Confidence intervals
MD: Mean difference
TCM: Traditional Chinese medicine.
