# Erratum to “Vitamin C as a Supplementary Therapy in Relieving Symptoms of the Common Cold: A Meta-Analysis of 10 Randomized Controlled Trials”

**DOI:** 10.1155/2021/9875417

**Published:** 2021-04-27

**Authors:** Li Ran, Wenli Zhao, Hongwu Wang, Ye Zhao, Huaien Bu

**Affiliations:** ^1^Graduate School, Tianjin University of Traditional Chinese Medicine, Tianjin 300193, China; ^2^Department of Occupational and Environmental Health, School of Health Sciences, Wuhan University, Wuhan 430071, China; ^3^Liver Center, Saga University Hospital, Saga University, 849-8501, Japan; ^4^School of Health Science and Engineering, Tianjin University of Traditional Chinese Medicine, Tianjin 300193, China; ^5^Qingdao Academy of Traditional Chinese Medicine, Shandong University of Traditional Chinese Medicine, Qingdao 266112, China

In the article titled “Vitamin C as a Supplementary Therapy in Relieving Symptoms of the Common Cold: A Meta-Analysis of 10 Randomized Controlled Trials” [1], author Huaien Bu was not included as a corresponding author by mistake. The correct version of the correspondence details is shown above. This mistake was introduced during the production of the article, and the publisher apologizes for this error.
